# Gut-microbiota prompt activation of natural killer cell on alcoholic liver disease

**DOI:** 10.1080/19490976.2023.2281014

**Published:** 2023-11-21

**Authors:** Jung A Eom, Jin-Ju Jeong, Sang Hak Han, Goo Hyun Kwon, Kyeong Jin Lee, Haripriya Gupta, Satya Priya Sharma, Sung-Min Won, Ki-Kwang Oh, Sang Jun Yoon, Hyun Chae Joung, Kyung Hwan Kim, Dong Joon Kim, Ki Tae Suk

**Affiliations:** a Institute for Liver and Digestive Diseases, Hallym University, Chuncheon, Republic of Korea; b Department of Pathology, Hallym University College of Medicine, Chuncheon, Republic of Korea; c Chong Kun Dang Bio Research Institute, Ansan-si, Gyeonggi-do, Republic of Korea

**Keywords:** Alcoholic liver disease, immune, NK cell, gut microbiota, gut liver axis

## Abstract

The liver is rich in innate immune cells, such as natural killer (NK) cells, natural killer T cells, and Kupffer cells associated with the gut microbiome. These immune cells are dysfunctional owing to alcohol consumption. However, there is insufficient data on the association between immune cells and gut microbiome in alcoholic liver disease (ALD). Therefore, the purpose of this study was to evaluate the effects of probiotic strains on NK cells in ALD patients. In total, 125 human blood samples [control (*n* = 22), alcoholic hepatitis (*n* = 43), and alcoholic cirrhosis (*n* = 60]) were collected for flow cytometric analysis. C57BL/6J mice were divided into four groups (normal, EtOH-fed, and 2 EtOH+strain groups [*Phocaeicola dorei* and *Lactobacillus helveticus*]). Lymphocytes isolated from mouse livers were analyzed using flow cytometry. The frequency of NK cells increased in patients with alcoholic hepatitis and decreased in patients with alcoholic cirrhosis. The expression of NKp46, an NK cell-activating receptor, was decreased in patients with alcoholic hepatitis and increased in patients with alcoholic cirrhosis compared to that in the control group. The number of cytotoxic CD56dimCD16^+^ NK cells was significantly reduced in patients with alcoholic cirrhosis. We tested the effect of oral administration *P. dorei* and *L. helveticus* in EtOH-fed mice. *P. dorei* and *L. helveticus* improved liver inflammation and intestinal barrier damage caused by EtOH supply and increased NK cell activity. Therefore, these observations suggest that the gut microbiome may ameliorate ALD by regulating immune cells.

## GRAPHICAL ABSTRACT

**Figure:**
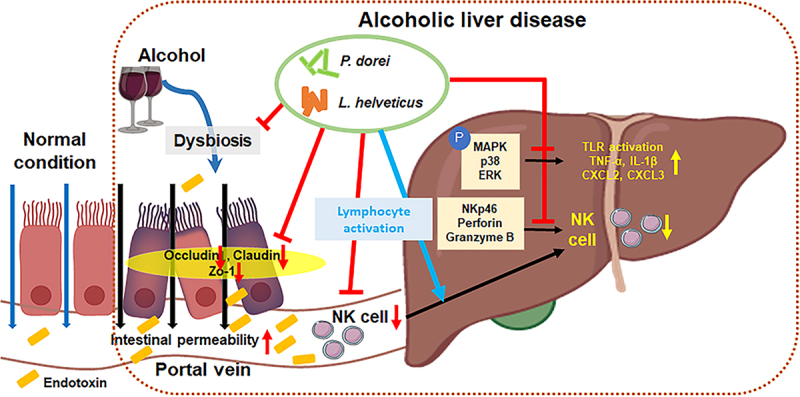

## Introduction

Excessive or chronic alcohol consumption can lead to a number of diseases, including alcohol use disorder, alcoholic liver disease (ALD), neurological disease, and immunologic disease.^1^ ALD is a common type of liver disease and its spectrum ranges from fatty liver characterized by fat accumulation in hepatocytes due to alcohol consumption and alcoholic steatohepatitis with immune cell infiltration and hepatocyte damage, to cirrhosis and hepatocellular carcinoma (HCC).^2^

It is known that ethanol-related metabolites (acetaldehyde and acetate) and oxidative stress generated during alcohol metabolism are main causes contributing to ALD.^3,4^ Chronic inflammation is a driving force of progression of ALD.^2^ Mechanisms for the onset and progression of ALD have not been fully elucidated yet. However, there is evidence that immune cells play an important role in inflammatory processes, such as repair of damaged liver, inflammation, and initiation of immune response.^5,6^

The liver is not only the main site of toxin elimination and nutrient metabolism but also an immune organ rich in various types of resident immune cells, such as Kupffer cells (KC), unconventional T cells, natural killer (NK) cells, and natural killer T (NKT) cells, which are innate lymphocytes.^7–9^ The liver can also rapidly recruit circulating immune cells.^10^ Immune cells in a damaged liver due to excessive fat accumulation and alcohol consumption contribute to the occurrence and development of ALD.^11^

Natural killer (NK) cells are present in various organs and tissues. They account for approximately 30–50% of all lymphocytes, especially in the liver.^12,13^ NK cells with a wide range of receptors for cytokines are immediately recruited and activated by various cytokines to infected tissues, increasing their anti-infective and anti-tumor abilities.^13,14^ They also play an anti-fibrotic role by promoting apoptosis through production of interferon gamma (IFN-γ) and directly killing hepatic stellate cells (HSCs).^15,16^ However, it is known that the number and function of NK cells may be decreased due to ethanol and its metabolites during chronic alcohol intake.^2,17^

Alcohol consumption may alter the composition of the gut microbiome. Accumulating evidence suggests that dysbiosis is a risk factor for ALD progression and development.^11^ Ethanol-induced increase in intestinal permeability causes bacterial and metabolite translocation, leading to inflammation and recruitment of circulating immune cells to the liver. It can also affect the functions of liver-resident immune cells.^18,19^

Probiotic therapy for the repair of gut microbiota and intestinal barrier damage might help inhibit the progression of ALD.^11^ Among different probiotics, *Lactobacillus* sp. have been shown to be effective in ameliorating liver inflammation.^20,21^ However, the immunological mechanism of ALD is not yet known in detail. The stool analysis results of patients with ALD showed that *Phocaeicola dorei* and *L. helveticus* increased with the progression of ALD. Several studies have shown that *P. dorei* reduces intestinal microbial lipopolysaccharide (LPS) production, inhibits atherosclerosis, and downregulates both local and systemic inflammatory responses.^22,23^ *L. helveticus* is known to modulate the host immune response and alleviate acute liver injury.^21,24,25^ Based on this knowledge, we hypothesized that *P. dorei* and *L. helveticus* could compensate for ALD by attenuating inflammation and modulating the immune response. Therefore, in this study, we evaluated the effects of *P. dorei* or *L. helveticus* on ALD. In addition, the effect of the strains on alcohol-induced reduction of NK cell activity was investigated.

## Results

### Liver function, triglyceride, cholesterol, and creatine level of human

Aspartate transaminase (AST), alanine transaminase (ALT), gamma-glutamyl transpeptidase (γ-GTP), and triglyceride (TG) levels were increased, whereas cholesterol and high-density lipoprotein (HDL) levels were decreased in ALD patients compared to healthy controls. There was no significant difference in creatine levels between patients with ALD and healthy controls (Supplementary Figure S1).

### Function and activity of NK cells change depending on the degree of progression of alcoholic liver disease

**Figure 1.**
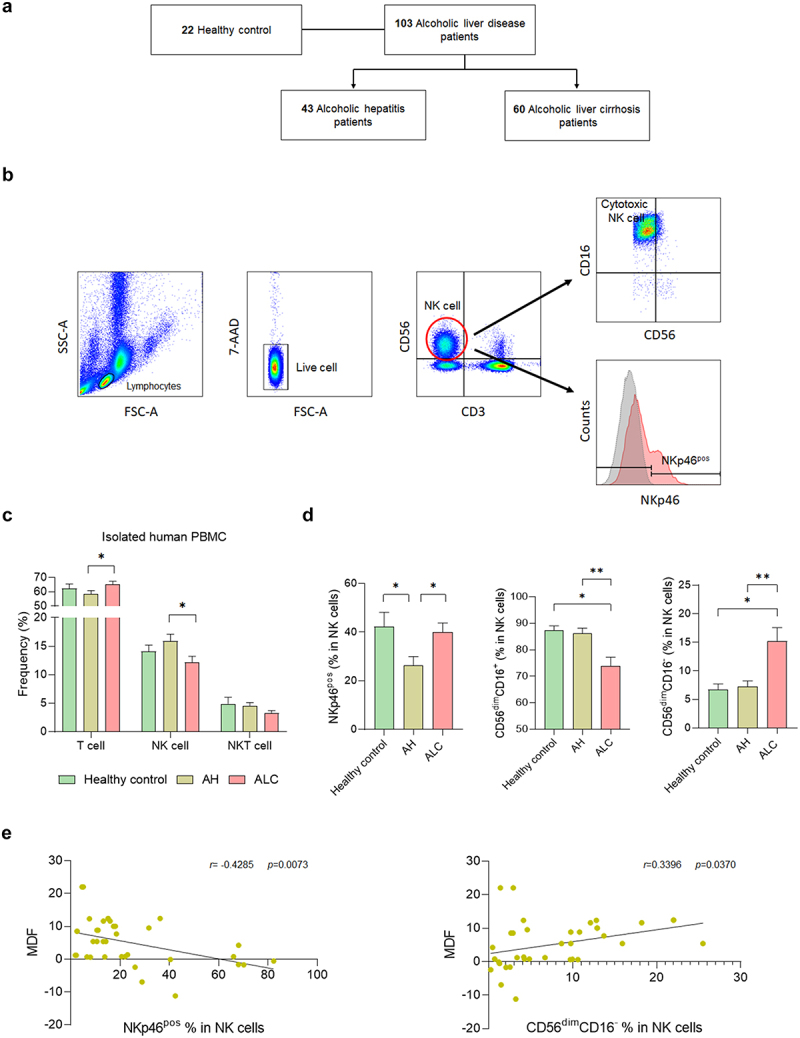
NK cell frequency and function change with the progression of alcoholic liver disease. (a) flow diagram. (b) flow cytometry analysis gating strategy. (c) flow cytometry analysis of PBMCs isolated from human blood. (d) frequency of NK cell activation receptor (NKp46) and NK cell with cytotoxic function in CD3^−^CD56^+^ NK cells. (e) correlation between alcoholic hepatitis severity score, MDF score, and NK cell function. Data are presented as the mean ± SEM. *p < .05, **p < .01 compared to corresponding controls using unpaired t test between the two groups. Abbreviations: ALD, alcoholic liver disease; AH, alcoholic hepatitis; ALC, alcoholic liver cirrhosis; MDF, maddrey’s discriminant function.

For the immune cell isolation, the peripheral blood mononuclear cells (PBMCs) were isolated from the blood of a total of 125 subjects, including 22 healthy controls, 43 patients with alcoholic hepatitis (AH, *n* = 43), and 60 patients with alcoholic cirrhosis (ALC, Figure 1a,b). Flow cytometry analysis showed that the frequency of T cells was significantly increased, whereas the frequency of NK cells was significantly decreased in ALC patients compared to AH patients (Figure 1c). There was no significant difference in the frequency of NKT cells between the two groups.

The activation and cytotoxicity of NK cells was investigated according to the degree of ALD. NKp46, an activating receptor for NK cells, was significantly decreased in AH patients compared to healthy controls but was significantly increased in ALC patients (Figure 1d). CD56^brt^CD16^+^ and CD56^brt^CD16^−^NK cells tended to increase in ALC; however, significant differences were not observed in any of the groups (Supplementary Figure S2a). To investigate the activation of NK cells according to the severity of hepatitis, changes in NKp46 in NK cells according to the Maddrey discriminant function (MDF) score, a disease prognostic measure in AH patients, were observed. The frequency of NKp46 in NK cells decreased when the MDF score increased, indicating a negative correlation. CD56^dim^CD16^−^ NK cells showed a positive correlation with MDF score (Figure 1e). However, CD56^dim^CD16^+^ NK cells did not show a significant correlation with MDF score (Supplementary Figure S2b). These data suggest that the number, activation, and cytotoxicity of NK cells can change depending on ALD progression.

### Alterations of human gut microbiome compositions in ALD

To determine changes in the gut microbiome of patients with ALD, 216 subjects were classified into four disease groups (Supplementary Figure S1). Subsequently, fecal metagenomic sequencing was performed.

**Figure 2.**
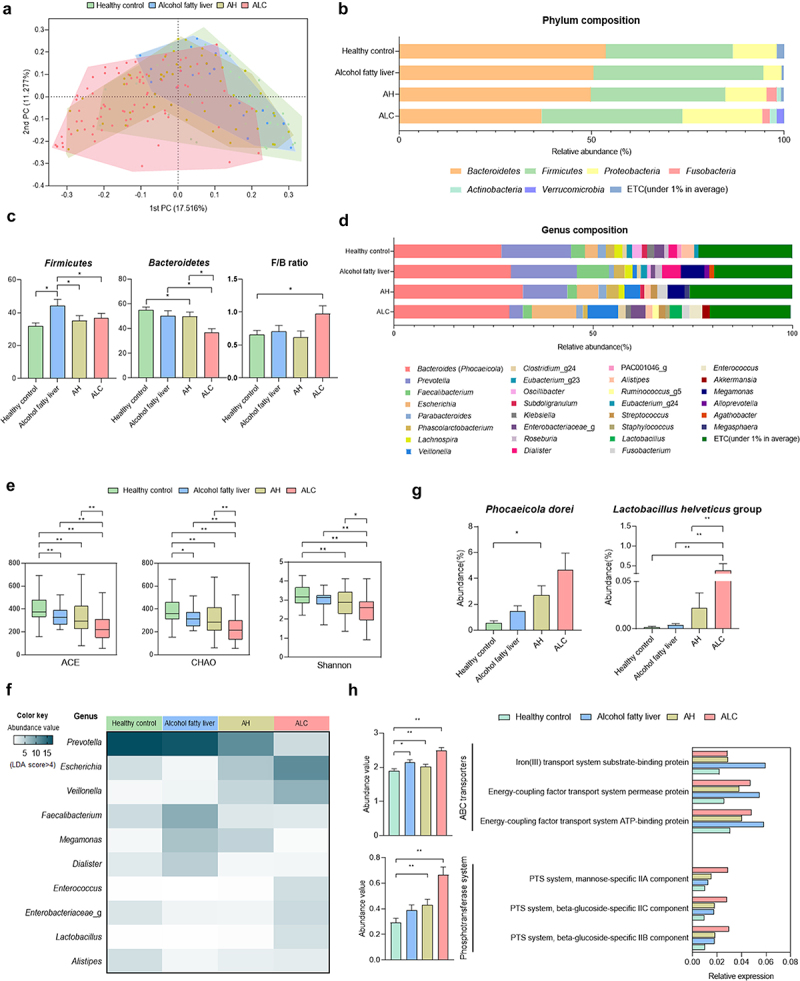
Comparisons of relative abundances of gut microbiome and functional biomarkers between healthy control and ALD patients. (a) beta diversity (principal coordinates analysis). (b) phylum composition of healthy control, alcohol fatty liver, AH, ALC patients. (c) the relative abundance of Firmicutes, Bacteroidetes and F/B ratio (%). (d) the genus composition of healthy control, alcohol fatty liver, AH, ALC patients. (e) alpha diversity through ACE and CHAO, which are species richness indicators, and Shannon analysis, which is species evenness indicators. (g) abundance of *P. dorei* and *L. helveticus* groups. (f) heatmap analysis with taxonomic biomarker LDE score > 4. (h) pathway and orthology analysis in functional biomarkers (LEfSe analysis). Data are presented as the mean ± SEM. *p < .05, **p < .01 compared to corresponding controls using unpaired t test between the two groups. Abbreviation: ACE, abundance-based coverage estimators; LDA, linear discriminant analysis; LEfSe, linear discriminant analysis effect size.

Beta diversity, an indicator of similarity between the microbiomes, showed differences between groups, although there was an overlap between disease groups (Figure 2a). *Firmicutes* and *Bacteroidetes* are the dominant phyla, accounting for more than 90% of the total population.^26^ At the phylum level, *Firmicutes* increased and *Bacteroidetes* decreased in patients with ALD. *Bacteroidetes* decreased as ALD progressed. F/B ratios are associated with several pathological conditions.^27^ These F/B ratios were significantly higher in ALC patients than in healthy controls. (Figure 2b,c).

At the genus level, the composition ratio of *Bacteroides* (some species were reclassified as the genus *Phocaeicola*) was higher in ALD patients than in healthy controls. In contrast, the composition ratio of *Prevotella* decreased as ALD progressed (Figure 2d). ACE, CHAO, and Shannon indices of alpha-diversity, showing cluster diversity within a sample, were analyzed. ACE and CHAO indicate species richness. Shannon index represents the evenness of a species in a microbial community. Its value increased with uniformity. All three indicators decreased as the ALD progressed (Figure 2e). In addition, genera with a linear discriminant analysis (LDA) score of 4 or higher, representing the effect size of each abundant genus, were selected as taxonomic biomarkers. *Lactobacillus* levels were significantly increased in patients with ALC (Figure 2f). Furthermore, in terms of species composition, it was confirmed that *P. dorei* and *L. helveticus* groups were increased in ALD patients compared to those in healthy controls. These values increased as the severity of ALD increased (Figure 2g). Functional biomarker analysis revealed significant differences in ABC transporters and phosphotransferase system (PTS) in the KEGG pathway (Figure 2h).

### *Supplementation with* P. dorei *and* L. helveticus *ameliorates the progression of alcoholic liver disease*

**Figure 3.**
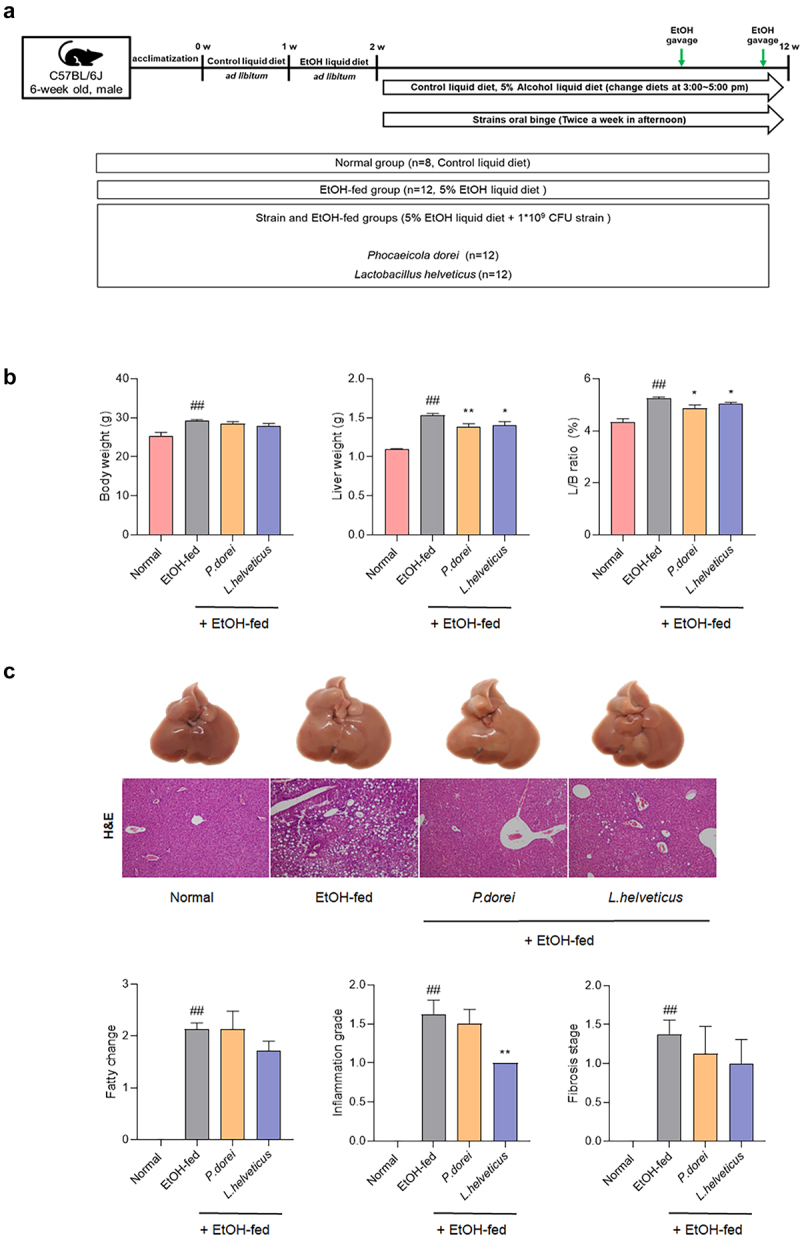
Results of histopathological changes in liver supplemented with *P. dorei* and *L. helveticus* in the chronic-plus-binge alcohol feeding model (NIAAA model). (a) scheme of the animal experiment. (b) changes in body weight, liver weight, and L/B ratio (%). (c) liver tissue pictures and H&E staining of liver tissue. Fatty change, inflammation grade, and fibrosis stage scores were evaluated through H&E-stained liver tissue. Data are presented as the mean ± SEM. #*p* < .05, ##*p* < .01 compared EtOH-fed and normal groups using an unpaired t test between the two groups. *p < .05, **p < .01 compared EtOH-fed and strain groups using an unpaired t test between the two groups.

Increased levels of *P. dorei* and *L. helveticus* were observed in ALD patients (Figure 2g). To evaluate whether *P. dorei* and *L. helveticus* could inhibit liver damage in an ALD model (NIAAA model), C57BL/6 mice were orally administered these strains in a 5% EtOH liquid diet for 10 weeks after undergoing an adaptation period of two weeks. To further induce inflammation, 31.5% ethanol was orally administered twice before sacrifice (Figure 3a). Body weight, liver weight, and L/B ratio were significantly higher in the EtOH-fed group than in the normal group. However, liver weight and L/B ratio were significantly decreased in the groups treated with *P. dorei* or *L. helveticus* (Figure 3b).

Histopathology results by H&E staining showed that feeding a 5% EtOH liquid diet for 10 weeks induced fatty changes, hepatitis, and fibrosis. In particular, administration of *L. helveticus* significantly reduced inflammation grade compared to the EtOH-fed group. Administration of *L. helveticus* also decreased the fatty change and fibrosis stage, although the decrease was not statistically significant. Administration of *P. dorei* did not significantly affect fatty changes. It only showed a tendency to decrease with inflammation grade and fibrosis stage. However, this decrease was not statistically significant (Figure 3c).

### Gut microbiome composition is altered by strain supplementation in the chronic-plus-binge alcohol feeding model (NIAAA model)

Microbiome taxonomic profiling (MTP) was performed using stool 16s rRNA sequencing to confirm changes in the gut microbiome in the NIAAA model mediated by ethanol diet and probiotic strain administration. Based on beta diversity, samples from the same group were close in distance from each other (Supplementary Figure S3a). ACE and Shannon indices were lower in the EtOH-fed group than in the normal group. In contrast, these cells recovered after treatment with *L. helveticus* (Supplementary Figure S3b).

At the phylum level, *Firmicutes* showed a decreasing trend in the *P. dorei* group, whereas *Bacteroidetes* increased in both the EtOH-fed group and the strains administered groups. The F/B ratio decreased in all other groups compared to that in the normal group (Supplementary Figure S3c and S3d). Differences in the abundance of the genera were also observed in each group (Supplementary Figure S3e).

Taxonomic biomarker analysis revealed that the genus composition was significantly different between groups (Supplementary Figure S3f). Five KEGG pathways were decreased in the EtOH-fed group compared with those in the normal group. These were recovered in *P. dorei* group (Supplementary Figure S3g). These results suggest that the gut microbiome can be altered when an ethanol diet and probiotic strains are administered. In addition, dietary changes in the gut microbiome can affect taxonomic biomarkers.

### P. dorei *and* L. helveticus *attenuate ethanol-induced liver damage by modulating inflammatory and immune response*

**Figure 4.**
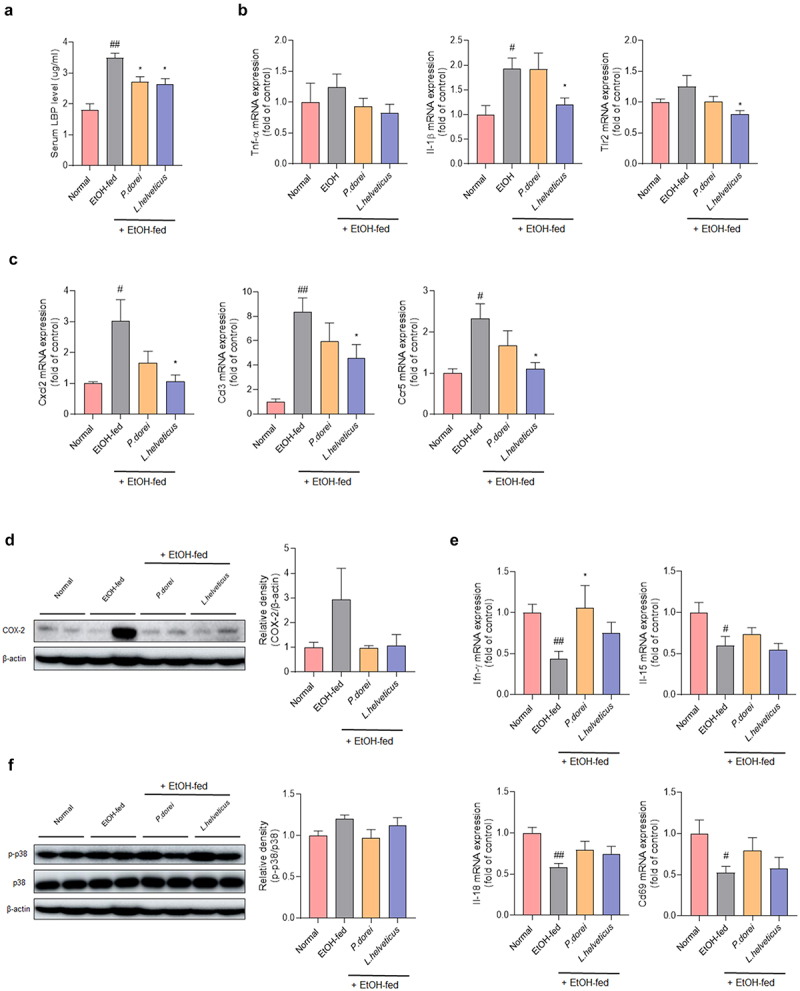
Effects of strains on expression of genes related to inflammation and immune response in chronic-plus-binge alcohol feeding model (NIAAA model) mouse liver. Liver tissue was collected and total RNA for RT-PCR analysis and protein for western blot were extracted. (a) LBP ELISA assay in mouse serum. Hepatic mRNA expression level of (b, e) pro-inflammatory cytokine and (c) chemokine genes that play an important role in immune and inflammatory control. Western blot analysis of the protein expression level of (d) COX-2 and (f) MAPK signaling pathway in liver tissue. Data are presented as the mean ± SEM. #*p* < .05, ##*p* < .01 compared EtOH-fed and normal groups using an unpaired t test between the two groups. *p < .05 compared EtOH-fed and strain groups using an unpaired t test between the two groups. Abbreviation: LBP, lipopolysaccharide binding protein; TNF-α, tumor necrosis factor α; IL, interleukin; TLR2, toll-like receptor 2; CXCL, C-X-C motif chemokine ligand; CCL, CC motif chemokine ligand; IFN-γ, interferon gamma; MAPK, mitogen-activated protein kinase.

Chronic alcohol administration can increase the levels of the endotoxin lipopolysaccharide (LPS) in the portal vein and serum. It is also known to contribute to alcohol-induced hepatitis. LPS can lead to the production of inflammatory cytokines by activating the TLR4 signaling pathway, binding to the LPS binding protein (LBP), and inducing an inflammatory response.^28,29^ Therefore, LBP level was assessed in the serum of mice. The LBP level was significantly higher in the EtOH-fed group than in the normal group. However, it was significantly decreased in the groups administered probiotic strains compared with the EtOH-fed group (Figure 4a).

Inflammatory cytokines were evaluated to determine the expression levels of genes related to ethanol-induced inflammatory responses in the mouse liver. TNF-α expression increased in the EtOH-fed group. It tended to decrease in the probiotic strain group. The expression of IL-1β was significantly increased in the EtOH-fed group compared to that in the normal group. It was decreased in the *L. helveticus* treated group compared with that in the EtOH-fed group.

It is known that chronic alcohol consumption can increase the expression of TLR2 and further enhance the expression of TNF-a in response to LPS.^30^ It was found that TLR2 was significantly decreased in the *L. helveticus* treated group compared to the EtOH-fed group (Figure 4b). The expression levels of CXCL2, CCL3, and CCR5 were significantly increased in the EtOH-fed group compared with those in the normal group. This was significantly recovered in the *L. helveticus* treated group (Figure 4c). A similar trend was observed in *P. dorei* treated group. In addition, COX-2 protein expression, which contributes to alcohol-induced liver inflammation, increased in the EtOH-fed group but decreased in the group treated with the probiotic strains (Figure 4d). IFN-γ is primarily produced by NK and NKT cells. It can promote NK cell activation.^31^ IL-15 induces NK cell proliferation. IL-18, also known as interferon-gamma-inducing factor, can induce an immune response.^32^ CD69 is expressed after NK cell activation. The expression level of IFN-γ was significantly decreased in the EtOH-fed group compared to that in the normal group. It was significantly higher in the *P. dorei* treated group than in the EtOH-fed group. A similar trend was observed for *L. helveticus* group. Levels of IL-15, IL-18, and CD69 were significantly lower in the EtOH-fed group than in the normal group. Most showed a tendency to recover following treatment with probiotic strains (Figure 4e).

It is known that chronic alcohol-induced phosphorylation of mitogen-activated protein kinase (MAPK) can induce liver damage.^33^ Therefore, the expression levels of MAPK family member proteins in liver tissues were analyzed. The results showed a tendency for p38 phosphorylation to increase in the EtOH-fed group but decrease in the group treated with the probiotic strains (Figure 4f). These results show that probiotic strains can improve ethanol-induced hepatitis by regulating inflammatory and immune responses.

### P. dorei *and* L. helveticus *can improve intestinal mucosa damage caused by ethanol*

**Figure 5.**
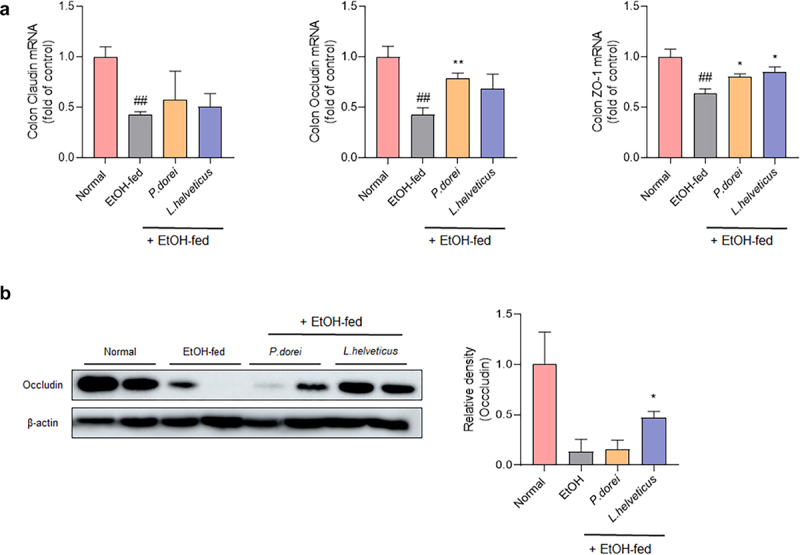
Effect of strains on recovery of impaired intestinal barrier caused by ethanol feeding in chronic-plus-binge alcohol feeding model (NIAAA model). Colon tissue was collected and total RNA for RT-PCR analysis and protein for western blot were extracted. (a) RT-PCR analysis and (b) western blot analysis of tight junction protein genes in mouse colon tissue. Data are presented as the mean ± SEM. ##*p* < 0.01 compared EtOH-fed and normal groups using an unpaired t test between the two groups. *p < .05, **p < .01 compared EtOH-fed and strain groups using an unpaired t test between the two groups.

Alcohol intake can increase intestinal permeability by causing damage to the intestinal mucosa. This allows harmful and potentially toxic endotoxins to enter the systemic circulation and contribute to alcoholic liver disease.^34^ Therefore, we evaluated whether the candidate probiotic strain could improve intestinal permeability (Figure 5). The results showed that claudin, occludin, and zo-1 mRNA expression levels were significantly decreased in the EtOH-fed group but increased in the group treated with a probiotic strain. Occludin expression significantly increased in *P. dorei* treated group. Zo-1 expression was significantly increased in both the *P. dorei* treated and *L. helveticus* treated groups (Figure 5a). Protein levels were evaluated by western blotting. As a result, it was confirmed that occludin protein levels decreased in the EtOH-fed group compared to the normal group but increased in the *L. helveticus* treated group (Figure 5b). These results suggest that probiotic strains can attenuate ethanol-induced intestinal epithelial barrier dysfunction.

### P. dorei *and* L. helveticus *activate NK cell*

**Figure 6.**
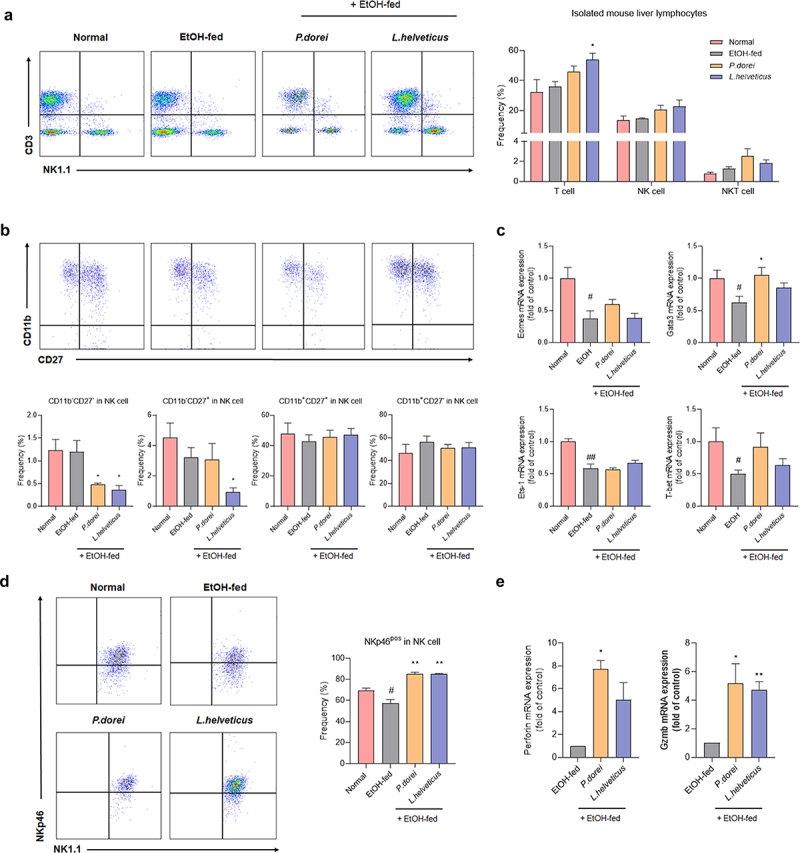
Effects of strains that increase the activity and function of NK cells that have been reduced by ethanol intake. (a) flow cytometry analysis of liver MNCs isolated from NIAAA model. (b) flow cytometry analysis of subsets of NK cells gated with NK1.1 and CD3. (c) hepatic mRNA expression levels of NK cell transcription factor genes. (d) frequency of activating receptor expression in NK cells gated with NK1.1 and CD3. (e) RT-PCR analysis of granzyme B and perforin in mouse liver NK cell. Data are presented as the mean ± SEM. #*p* < .05, ##*p* < .01 compared EtOH-fed and normal groups using an unpaired t test between the two groups. *p < .05, **p < .01 compared EtOH-fed and strain groups using an unpaired t test between the two groups.

To confirm the changes in immune cells caused by ethanol consumption and the effects of strains, lymphocytes were isolated from the mouse liver. The hepatic lymphocyte population was evaluated by fluorescence-activated cell sorting (FACS) analysis (Figure 6). T cells were significantly increased in *L. helveticus* treated group compared to those in the EtOH-fed group. The number of NK cells also increased in the group treated with the probiotic strain (Figure 6a).

Antibodies against CD27 and CD11b were used to examine the composition of NK cell subsets in CD3^−^NK1.1^+^ NK cells. The maturation of mouse NK cells starts at the CD11b^−^CD27^−^ stage and proceeds through the CD11b^−^CD27^+^ → CD11b^+^CD27^+^ → CD11b^+^CD27^−^. Immature CD11b^−^CD27^+^ NK cells are strong cytokine producers with weak cytotoxicity, whereas CD11b^+^CD27^+^ NK cells are highly cytotoxic to the liver and spleen. CD11b^+^CD27^−^ NK cells have limited cytotoxic ability.^35^

CD11b^+^CD27^+^ NK cells, known to exhibit predominant cytotoxicity, did not differ significantly between groups (Figure 6b). We also determined the mRNA expression levels of NK cell transcription factors. Gata-3, Eomes, and T-bet are essential for NK cell maturation. Eomes and T-bet also regulate granzyme B and perforin expression. Ets-1 is an important factor in the early development of NK cells. It is also essential for the expression of key receptors, such as NKp46, Ly49H, and Ly49D, which regulate NK cell activity.^36^ In the expression levels of all these transcription factors were significantly decreased. In the group treated with the probiotic strain, their levels tended to increase, although this increase was not statistically significant. Among them, Gata-3 expression was significantly increased in the *P. dorei* treated group. Gata-3 is also known to regulate the expression of T-bet.^37^ Our results showed that the expression patterns of Gata-3 and T-bet were consistent (Figure 6c). Although there was no difference in NK cell cytotoxicity, there was a difference in the expression of NK cell-activating receptors. NKp46 expression was significantly decreased in the EtOH-fed group compared to that in the normal group but increased significantly in the group treated with a probiotic strain (Figure 6d). In addition, perforin and granzyme B released by NK cells when lysing target cells were significantly increased in the group treated with the probiotic strain compared to those in the EtOH-fed group (Figure 6e). These results indicate that ethanol consumption can inhibit NK cell activation. However, treatment with probiotic strains can increase NK cell activation.

### Involvement of a probiotic strain in immune response is confirmed through hepatic RNA gene expression analysis

**Figure 7.**
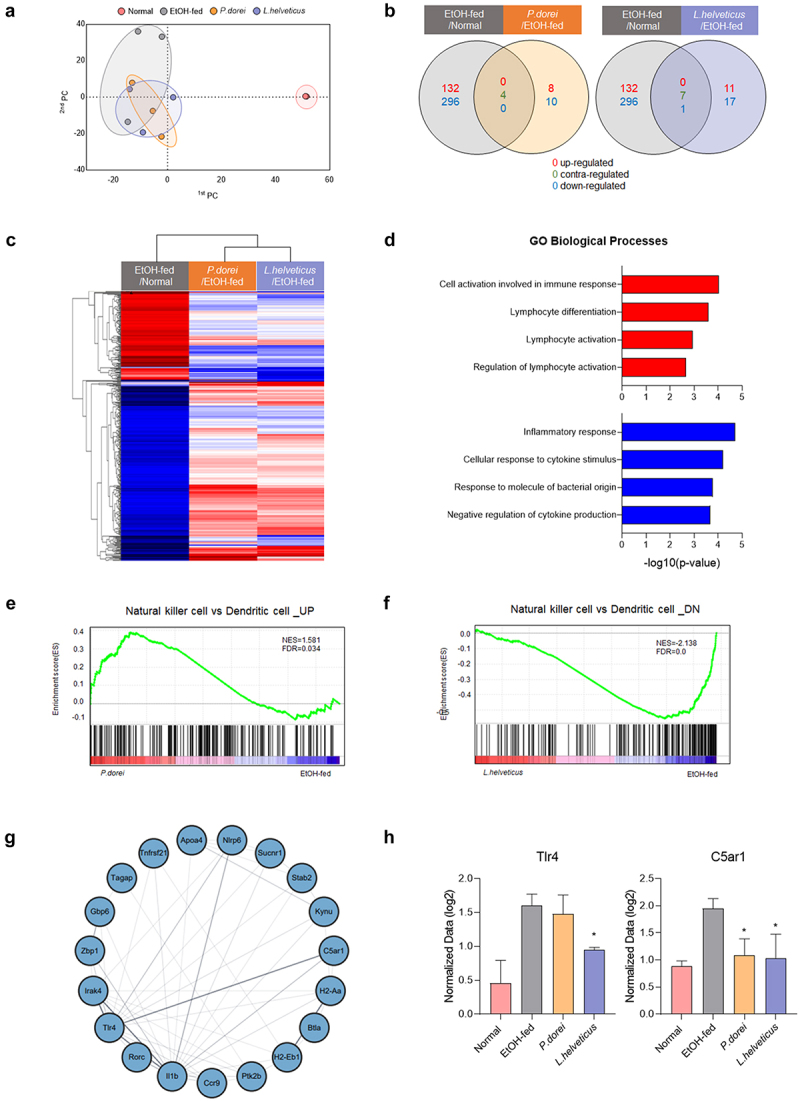
Results of QuantSeq mRNA-seq analysis in chronic-plus-binge alcohol feeding model (NIAAA model). (a) PCA based on the expression value of all genes in the sample. (b) venn diagram showing the difference in total gene expression between the EtOH-fed group and the strain group. (c) clustering heatmap analysis showing the similarity between samples and genes. (d) GO biological process analysis of the strain group compared to the EtOH-fed group using metascape. (e) GSEA analysis of upregulated gene expression in NK cells increased by *P. dorei* administration. (f) GSEA analysis of downregulated gene expression in NK cells reduced by *L. helveticus* administration. (g) Cytoscape STRING analysis of gene correlation based on protein-protein interaction. (h) genes involved in inflammation and immune response with significantly different expression. Abbreviation: PCA, principal component analysis; GO, gene ontology; NES, normalized enrichment score; FDR, false discovery rate.

Total RNA sequencing analysis was performed to investigate the differences in gene expression between the EtOH-fed group and the group treated with the probiotic strain (Figure 7). PCA analysis to determine the similarity between samples for all gene expressions showed that samples from the same group were similar (Figure 7a). Compared with the normal group, 132 genes were upregulated and 296 genes were downregulated in the EtOH-fed group. Eight genes were upregulated and 10 genes were downregulated in the *P. dorei* treated group compared with those in the EtOH-fed group. Four genes showed contrasting expression regulation in both groups. Compared to the EtOH-fed group, 11 genes were upregulated and 17 genes were downregulated in the *L. helveticus* treated group. Seven genes showed contrasting expression regulation in both groups, and one gene was downregulated (Figure 7b).

Clustering heat map analysis showed similarities between the two groups treated with a probiotic strain. It also revealed that genes up- or downregulated in the EtOH-fed group compared to the normal group showed contrasting regulation in the group treated with a probiotic strain (Figure 7c). Gene ontology (GO) biological processes were analyzed based on up- and downregulated immune response genes in the group treated with a probiotic strain compared to the EtOH-fed group. In the group treated with a probiotic strain, genes related to cell activation involved in the immune response and lymphocyte activation were upregulated, whereas genes related to the inflammatory response were downregulated (Figure 7d). Gene set enrichment analysis (GSEA) showed an increase in the number of upregulated genes in NK cells versus dendritic cells in the *P. dorei* treated group compared to that in the EtOH-fed group (Figure 7e). In addition, compared to the EtOH-fed group, the *L. helveticus* treated group showed a decrease in downregulated genes in NK cells compared to dendritic cells (Figure 7f). These results indicate that the probiotic strain is involved in the regulation of NK cell-related genes.

Cytoscape STRING analysis was performed to show correlations between genes based on protein – protein interactions. The results showed that the genes involved in the immune response interacted with each other (Figure 7g). In addition, among the genes involved in immune responses, it was confirmed that TLR4, which is activated by recognizing LPS^38,39^, and C5AR1, which regulates inflammatory response, obesity, and cancer development^40–42^, were increased significantly in the EtOH-fed group but decreased in the group treated with a probiotic strain (Figure 7h). These results demonstrate that probiotic strains can improve alcoholic liver disease by regulating inflammation and immune responses.

## Discussion

Over the past few years, considerable research has been conducted on the effects of the gut microbiota on host health. It has been shown that gut microbiome dysbiosis is associated with a variety of diseases. An increase in intestinal permeability due to damage allows bacteria and their components to enter blood and tissues and travel to the liver, thus affecting liver’s immune response.^43^ Our study confirmed changes in intestinal microbiota in ALD and investigated whether specific probiotic strains could improve ALD through the immunomodulatory ability of such probiotics.

To investigate the relationship between ALD and NK cells, we observed changes in NK cells in blood samples of patients with ALD. Compared to healthy controls, we found that NK cells were increased in patients with AH but decreased in patients with ALC. Additionally, NK cell activation decreased in patients with AH but increased in patients with ALC. In line with these findings, NK cell activity decreased when alcoholic hepatitis became more severe. In addition, the number of cytotoxic NK cells is greatly reduced in patients with ALC. Several studies have shown that alcohol can inhibit the immune system and regulate NK cell activity by suppressing the number and function of NK cells.^44–46^ Our results are consistent with the results of these studies. Taken together, these results suggest that the number and activity of NK cells can change with ALD progression.

Human stool 16s rRNA was analyzed to observe changes in the intestinal microflora according to the progression of ALD. As ALD progressed, species richness and evenness decreased. Changes in phylum and genus composition were also observed. Firmicutes levels were increased in the ALD group. *Bacteroidetes* decreased as alcoholic liver disease progressed. Several studies have shown that alcohol can reduce anti-inflammatory *Faecalibacterium* and *Roseburia*, which can affect gastric barrier integrity and increase opportunistic pathogens such as *Streptococcus* and *Enterococcus*.^47–49^ Other studies also suggest that *Veillonella* is increased in patients with AH and that *Lactobacillus* is increased with higher alcohol intake.^50^ Consistent with these studies, our results showed that *Faecalibacterium* and *Roseburia* decreased in AH and ALC patients, whereas *Streptococcus*, *Enterococcus*, *Veillonella*, and *Lactobacillus* increased. At the species level, *P. dorei* and *L. helveticus* groups increased as ALD progressed. We observed that *Bacteroidetes* decreased in ALD but *P. dorei* increased. This is thought to be a compensatory mechanism for ALD. There is evidence that *Bacteroides* (*Phocaeicola*) as beneficial bacteria can provide nutrients and vitamins to the host, and that *Lactobacillus* species can improve ALD.^51,52^ Therefore, we hypothesized that *P. dorei* and *L. helveticus* are increased to suppress ALD.

To test this hypothesis and the effect of the gut microbiome on NK cells in ALD, ethanol-fed mice were orally administered *P. dorei* and L. helveticus. Studies have shown that *P. dorei* can reduce intestinal microbial LPS production and inhibit atherosclerosis.^22^ *L. helveticus* can alleviate liver damage caused by D-galactosamine by reducing inflammation.^24^ In our study, supplementation with *P. dorei* and *L. helveticus* reduced ethanol-induced hepatitis. In particular, *L. helveticus* effectively suppressed the inflammation.

The endotoxin LPS, a major factor in the development of alcoholic liver disease, is transported to the liver by increased intestinal permeability. It can cause inflammation, activate innate immune cells, and release various pro-inflammatory cytokines and chemokines.^53^ LPS binding protein (LBP) can bind to LPS and present it to TLR4 to elicit an immune response. Serum LBP levels were significantly elevated in the EtOH-fed group. However, it was effectively decreased by treatment with the probiotic strain. *L. helveticus* reduced TLR2 expression. Pro-inflammatory cytokines TNF-a and IL-1b, chemokines CXCL2 and CCL3, and chemokine receptor CCR5 were significantly decreased in the group treated with the probiotic strain compared to those in the EtOH-fed group. The cytokines IFN-γ, IL-15, and IL-18 can induce immune responses by promoting NK cell activation and proliferation. They were significantly lower in the EtOH-fed group than in the normal group. They increased in the group treated with a probiotic strain, although the increase was not statistically significant. Among them, IFN-γ levels were significantly increased in the *P. dorei* treated group. These findings confirm the role of probiotics in reducing ethanol-induced inflammation and inducing NK cell activation.

As mentioned earlier, experiments were conducted to demonstrate alcohol-induced intestinal barrier damage and the effects of probiotic strains. In the EtOH-fed group, the expression levels of claudin, occludin, and zo-1 mRNAs were all significantly decreased. Such a decrease in tight junction proteins is evidence of increased intestinal permeability.^54^ However, occludin and zo-1 mRNA expression levels increased significantly in the group treated with a probiotic strain. An increase in the protein level was also observed. These results suggest that the probiotic strain can improve intestinal barrier damage caused by alcohol by increasing tight junction proteins.

Considering that NK cells constitute the dominant lymphocyte population in the liver and that alcohol can suppress these NK cells^55^, we conducted an experiment to determine the changes in NK cells caused by probiotic strains in ALD. There were no significant differences in NK cell frequency or cytotoxicity in mice with ethanol-induced liver disease. Gata-3, Eomes, T-bet, and Ets-1 are transcription factors that are involved in the maturation and development of NK cells. The expression levels of these proteins were examined. The expression levels of these transcription factors were significantly decreased in the EtOH-fed group but increased in the group treated with a probiotic strain, although the increase was not statistically significant. The expression of Gata-3 was significantly decreased in the EtOH-fed group but increased in the *P. dorei* treated group. Another study demonstrated that Gata-3 is involved in NK cell maturation and that the number of NK cells in the liver of Gata-3-/- mice is rapidly reduced, suggesting that Gata-3 is involved in regulating NK cell homeostasis.^37^ However, as mentioned above, in our study, there was no significant difference in the frequency or maturation of NK cells between the EtOH-fed group and the group treated with a probiotic strain.

In our study, NK cell activity was altered in the mouse liver. The NK cell activation receptor NKp46 was significantly decreased in the EtOH-fed group, but significantly increased in the group treated with the probiotic strain. This decrease in NK cell activation in the EtOH-fed group was consistent with that found in patients with AH. In addition, there is evidence that granzyme B and perforin are released when NK cells are activated.^56^ Our results showed that, similar to NKp46 receptor expression, granzyme B and perforin expression levels were significantly increased in the group treated with a probiotic strain.

CD11b^+^CD27^+^ NK cells have a dominant cytotoxic effect on the liver. They showed a tendency to decrease in the EtOH-fed group and increase in the group treated with a probiotic strain, although this increase or decrease was not statistically significant. In addition, CD11b^+^CD27^−^ NK cells, the next maturation stage, tended to increase in the EtOH-fed group, but decreased in the group treated with a probiotic strain. These results suggest that the activity of NK cells is decreased due to ethanol supply and that their cytotoxic function is not properly performed, leading to progression to the next mature stage. However, probiotic treatment activated NK cells and increased the expression of perforin and granzyme B. Because it increased cytotoxicity and killed HSC activated by ethanol, the next maturation stage did not progress compared with the EtOH-fed group. Thus, CD11b^+^CD27^−^ NK cells in the group treated with the probiotic strain were relatively lower than those in the EtOH-fed group. Further research is needed to confirm this hypothesis. Therefore, probiotic strains can enhance NK cell activity in experimental ALD livers.

Sequencing analysis of total RNAs isolated from liver tissues revealed that the group treated with a probiotic strain showed upregulation of genes involved in lymphocyte activation and cell activation in the immune response and downregulation of genes involved in the inflammatory response compared to the EtOH-fed group. In addition, the number of upregulated genes in NK cells increased in the *P. dorei* treated group, and the number of downregulated genes decreased in the *L. helveticus* treated group. Among the genes involved in various inflammatory and immune responses, TLR4 and C5AR1 were increased in the EtOH-fed group but decreased in the group treated with a probiotic strain. TLR4 is a receptor that can induce an inflammatory response by binding to LPS.^57^ One study has shown that C5AR1 can induce colorectal tumorigenesis by controlling tumor-promoting immune responses.^58^ These results demonstrate that the probiotic strains tested in this study can regulate inflammation and immune responses.

In conclusion, *P. dorei* and *L. helveticus* attenuated the intestinal microbial imbalance and intestinal and hepatic inflammation in ALD. Supplementation with *P. dorei* and *L. helveticus* also improved ALD by increasing NK cell activity, which was reduced by alcohol. Therefore, additional research is needed to identify the mechanisms involved in the control of immune response by these probiotic strains in ALD.

ALD is the most common liver disease in the world, has a high mortality rate, requires liver transplantation for definitive treatment, and has no fundamental cure. Considering that alcohol impairs gut barrier function and reduces NK cell activity, treatment with probiotic strains reverses these alcohol-induced defects, which may have important clinical implications in the treatment of alcoholic liver disease. Therefore, future clinical trials will be needed because these probiotic strains can help develop personalized immune treatments for individual patients.

## Materials and methods

### Patients

This prospective cohort study was conducted between April 2017 and March 2020 (ClinicalTrials.gov NCT04339725). This study included patients with liver disease who were followed up by the Department of Liver Disease. A total of 224 subjects, including healthy controls (*n* = 42), alcoholic fatty liver (*n* = 29), alcoholic hepatitis (AH, *n* = 61), and alcoholic cirrhosis (ALC, *n* = 84), were enrolled and analyzed. The healthy control group was enrolled as a normal person who visited the hospital for a health checkup. Regardless of study enrollment, patients received standard care for their disease. Exclusion criteria were a history of viral hepatitis, nonalcoholic fatty liver disease, autoimmune hepatitis, pancreatitis, hemochromatosis, Wilson’s disease, cancer, or drug-induced liver damage.

This study was governed by the ethical guidelines of the 1975 Declaration of Helsinki and approved by the Institutional Review Notice for Human Studies of hospitals participating in clinical trials (2016–134). Informed consent for registration was obtained from all participants.

Baseline assessments of complete blood counts, liver function tests, and viral markers were performed. Patients with AH and ALC underwent abdominal ultrasonography or CT. AST, ALT, creatine, cholesterol, γ-GTP, TG, and high-density lipoprotein (HDL) cholesterol were included as serum biochemical parameters. The enrolled patients and controls underwent stool sampling and clinical analysis. The clinical data were consistent with the metagenomic data. In the control group, stool samples were collected during health checkups, and in patients, stool boxes were collected and stored in a refrigerator at −80°C during hospitalization.

### Isolation of human peripheral blood mononuclear cells (PBMCs)

All blood samples were collected from the outpatient clinic with permission. The collected blood samples were analyzed daily. Whole blood was carefully layered on Histopaque-1077 solution and centrifuged at 400 × g for 30 min at room temperature. After centrifugation, the upper layer was carefully discarded with a pipette, and the PBMC layer was transferred to a new 15 ml tube. The samples were washed by centrifugation at 250 × g for 10 min using phosphate-buffered saline (PBS) solution. The supernatant was discarded, and the cell pellet was resuspended in PBS and centrifuged under the same conditions. After repeating this process once more, the supernatant was discarded, and the cell pellet was resuspended in ACK lysis buffer to remove red blood cells, incubated for 3 min, and centrifuged. The supernatant was discarded and a cell pellet containing lymphocytes was obtained.

### Animal chronic-plus-binge alcohol feeding model (NIAAA model)

Six-week-old specific-pathogen-free C57BL/6J male mice were purchased from Doo-Yeol Biotech (Seoul, Korea). Mice were housed periodically and housed in an animal facility under controlled conditions at 22 ± 2°C, 12/12-hour light/dark cycles, and humidity ranging from 40–50%. The mice had ad libitum access to food and water for the duration of the experiment. All procedures were performed in accordance with the National Institute of Health Guidelines for the Care and Use of Laboratory Animals. Before starting the NIAAA model, mice were acclimated to a chow diet for one week. They were then given an isocaloric control liquid diet for one week and had an adaptation period to the liquid diet. After the adaptation period, the mice were fed either an isocaloric control liquid diet or a Lieber-DeCarli liquid diet containing 5% ethanol for 10 weeks. Lieber-DeCarli was purchased from Doo-Yeol Biotech (Seoul, Korea).

Mice were orally administered *P. dorei* or *L. helveticus* at a dose of 109 CFU three times a week. Two weeks and one day before sacrifice, mice were orally administered ethanol (5 g ethanol/kg per kg of body weight). All procedures were approved by the institutional review board. Animal Care and Use Committee of the College of Medicine, Hallym University (Hallym 2021–64).

### Quantitative real time polymerase chain reaction

Total RNA was extracted using TRIzol Reagent (Invitrogen). RNA purity was measured using a spectrophotometer, and RNA was reverse-transcribed into cDNA using a high-capacity cDNA reverse transcription kit (Applied Biosystems). RT-PCR was performed using the PowerUp SYBR Green Master Mix (Applied Biosystems) and primers. Primers used are listed in Supplementary Table s1.

### Western blot

Liver tissue was homogenized in RIPA buffer (Thermo Scientific). Homogenized liver tissue was then incubated on ice for 1 h and then centrifuged at −4°C for 10 min at 14,000 RPM. The collected supernatant was quantified using BCA protein assay kit (Thermo Scientific) and stored at −20°C. Proteins were diluted in protein sample buffer (Elpis Biotech), electrophoresed on an SDS polyacrylamide gel, and transferred to a PVDF membrane. Membranes were blocked using 5% BSA and 5% skim milk in TBST (1X Tris buffered saline, 0.1% Tween 20) at room temperature for 1 h to prevent nonspecific binding. Primary antibodies (Abcam, 1:1000, Anti-COX2/Cyclooxygenase 2 antibody (ab15191); Cell signaling, 1:1000, p38 MAPK (D13E1) #8690; Cell signaling, 1:1000, Phospho-p38 MAPK (Thr180/Tyr182) (D3F9) #4511;Cell signaling, 1:1000, p44/42 MAPK (Erk1/2) (137F5) #4695;Cell signaling, 1:2000, Phospho-p44/42 MAPK (Erk1/2) (Thr202/Tyr204) (D13.14.4E) #4370; Abcam, 1:500, Anti-Occludin antibody [EPR8208] (ab167161); Abcam, 1:200, Anti-Claudin 1 antibody (ab15098)) incubated overnight at 4°C. The sections were washed three times for 5 min with TBST and incubated for 1 h at room temperature with a secondary antibody diluted 1:10000. The membrane was then washed three times for 5 min each with TBST. To visualize the protein bands, the membrane was reacted with Immobilon Forte Western HRP (MERCK) substrate solution for 2 min and analyzed using an Amersham imager 680 machine. Data analysis was performed using ImageJ software.

### Isolation of hepatic immune cells (liver mononuclear cell isolation) and NK cell

After sacrifice, the liver tissue was collected and minced in a 70-um cell strainer. Primary murine liver mononuclear cells (MNCs) were isolated by centrifugation using a density gradient of 40% Percoll (Sigma-Aldrich). NK cells were obtained using a FACS AriaII (flow cytometry sorter).

### Flow cytometry analyzes and fluorescence-activated single cell sorting (FACS)

After the isolation of human PBMCs and mouse liver monocytes and staining with various antibodies, human PBMCs were analyzed using a BD FACS Canto flow cytometer, and mouse liver monocytes were sorted and analyzed using a FACS AriaII. The antibodies used are listed in Supplementary Table S2.

### Serum endotoxin level analysis

Serum samples were collected from 18-week-old control and treated mice. Serum samples were centrifuged at 2000 × g for 20 min overnight at 2–8°C. After centrifugation, the supernatant was collected and stored at −80°C, and serum endotoxin levels were measured using the Mouse LBP SimpleStep ELISA Kit (ab269542, Abcam) according to the manufacturer’s instructions.

### QuantSeq 3‘mRNA-sequencing

Total RNA sequencing analysis extracted from mouse liver tissue was performed by eBiogen, Inc. (Seoul, South Korea). Gene ontology (GO), differentially expressed genes (DEG), clustering heatmap, and principal component analysis were performed using the ExDEGA (Excel based Differentially Expressed Gene Analysis) tool and ExDEGA Graphic Plus provided by eBiogen Inc. (Seoul, South Korea). Gene correlation analysis based on the Protein-Protein Interaction database was performed using the Cytoscape STRING tool (https://string-db.org/).

### Statistical analysis

Statistical analyses were performed using GraphPad Prism version 8 (Graph Pad, San Diego, CA). Comparisons between groups were analyzed using the – Mann Whitney U test, a non-parametric equivalent t-test. All data are expressed as the standard error of the mean (SEM) and were considered significant when *p* < .05.

## Supplementary Material

Supplementary file.docx

## Data Availability

The authors confirm that the data supporting the findings of this study are available within the article and its supplementary materials.
