# Supplementary material for: Gut-microbiota prompt activation of natural killer cell on alcoholic liver disease
Source: Gut Microbes. 2023 Nov 21;15(2):2281014. doi: 10.1080/19490976.2023.2281014 (PMC10730232; doi:10.1080/19490976.2023.2281014)
Supplement: Supplementary file.docx [file KGMI_A_2281014_SM4707.docx]

**Supplementary file**

**Gut-microbiota prompt activation of natural killer cell on alcoholic liver disease**


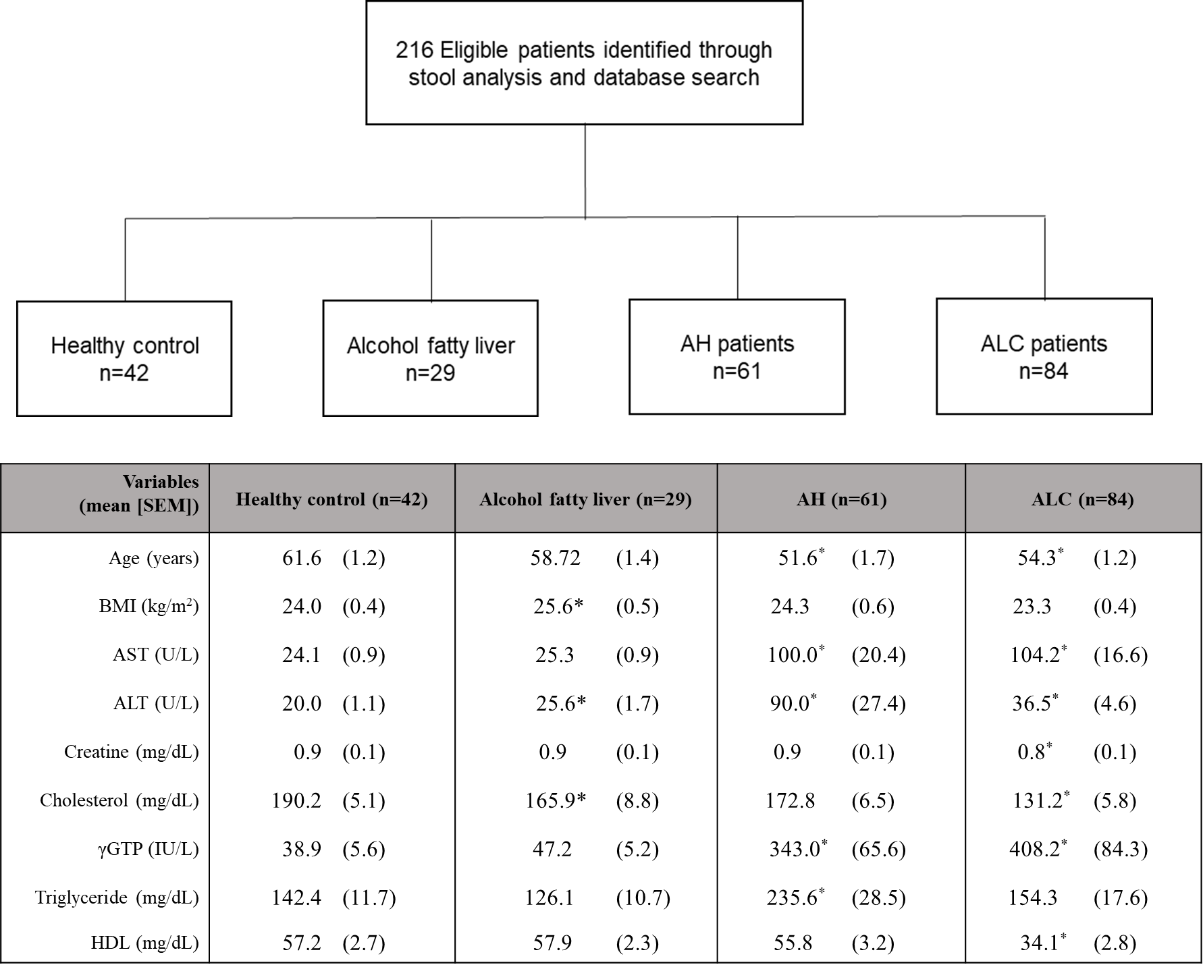


**Supplementary Figure 1. Baseline characteristics of patients**

***^
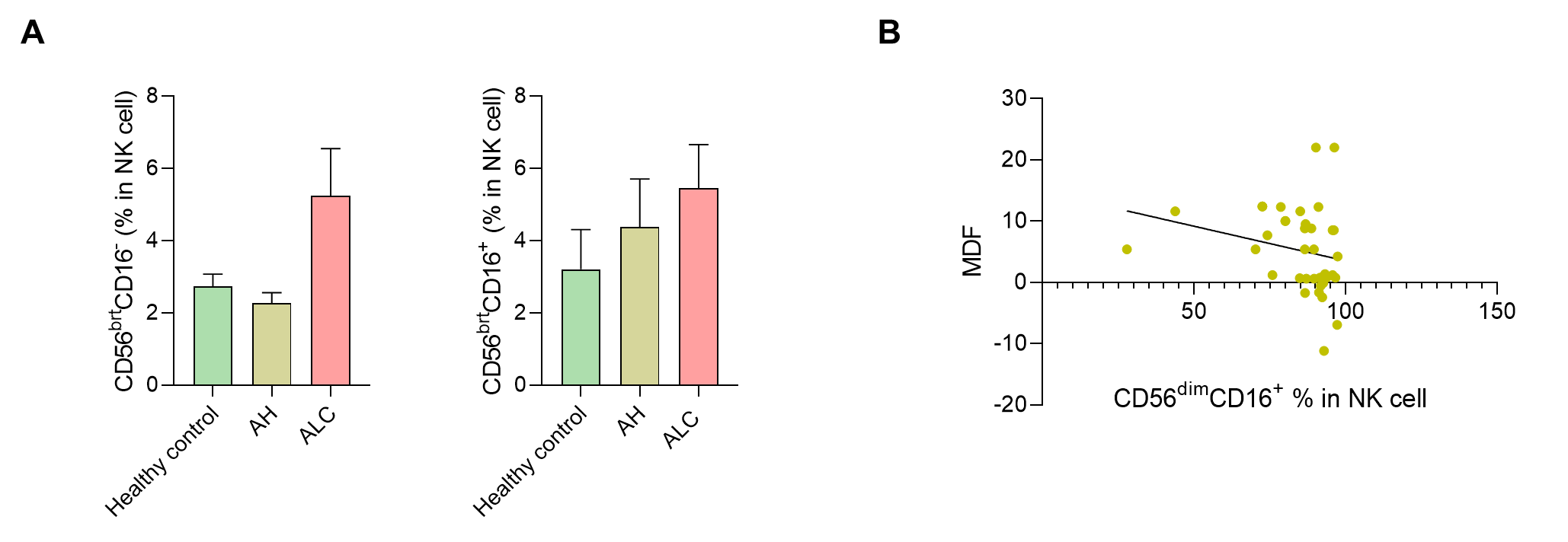
^***

**Supplementary Figure 2. Subpopulation frequency of CD3-CD56+ NK cells. Correlation between alcoholic hepatitis severity score, MDF score, and cytotoxic NK cells**


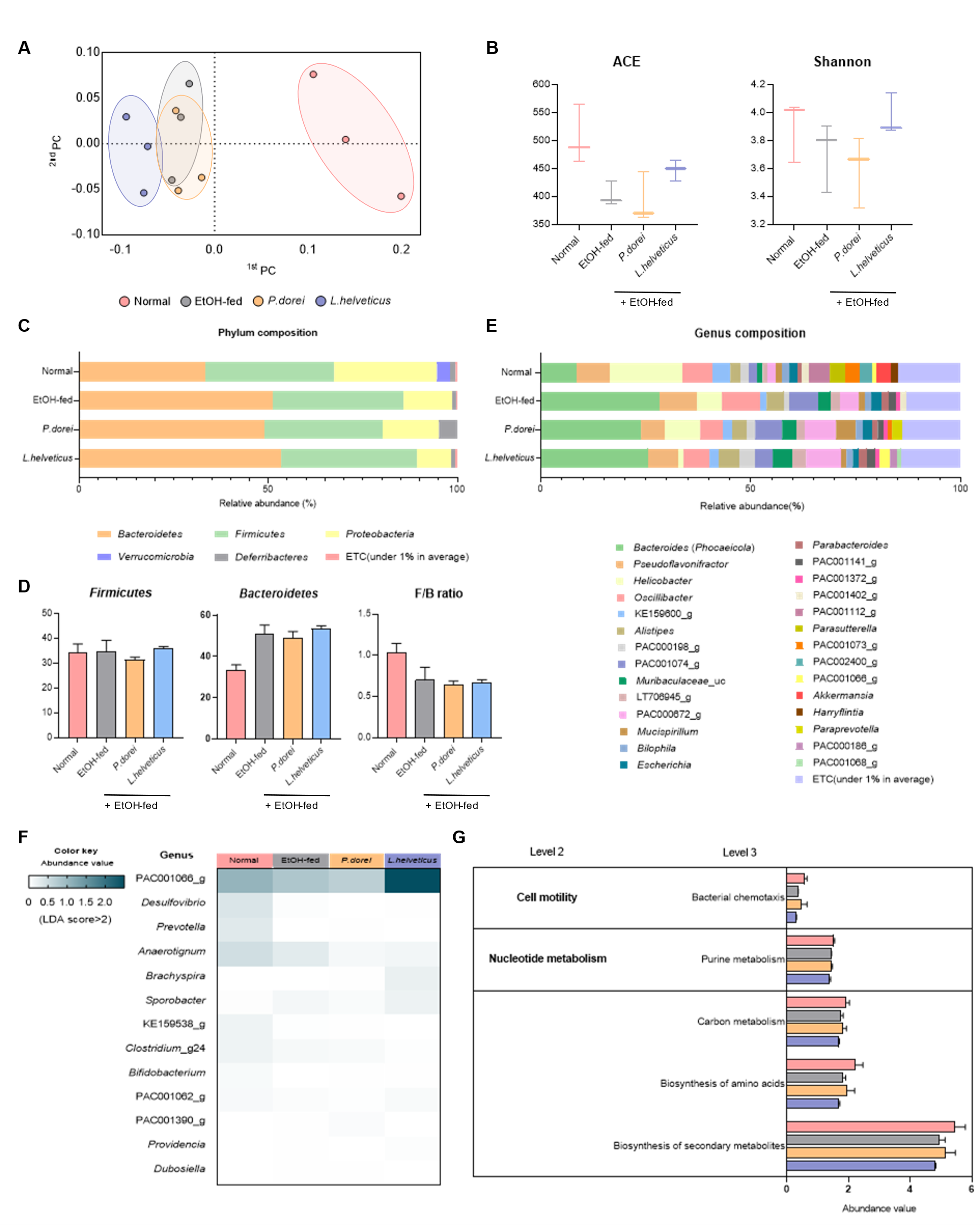


**Supplementary Figure 3. Mouse stool analysis in the NIAAA model.**

(A) Beta diversity (Principal coordinates analysis). (B) Alpha diversity analysis through ACE and Shannon showing species richness and evenness.

(C) Phylum and (D) genus composition. (E) Abundance of Firmicutes and Bacteroidetes and Ratio of Firmicutes to Bacteroidetes.

(F) Heatmap analysis with toxonomic biomarker LDE score >2. (G) Pathway analysis in functional biomarkers (LEfSe analysis). Data are presented as the mean ± SEM.

Abbreviation: ACE, Abundance-based coverage estimators; LDE, Linear discriminant analysis; LEfSe, Linear discriminant analysis effect size.

**Supplementary Table 1. Primers used in this study.**

| **Gene name** | **Forward sequence (5'-> 3')** | **Reverse sequence (5'-> 3')** |
| --- | --- | --- |
| TNF-a | CCCCAAAGGGATGAGAAGTT | CACTTGGTGGTTTGCTACGA |
| IL-1b | TGTGAAATGCCACCTTTTGA | GGTCAAAGGTTTGGAAGCAG |
| TLR2 | ACAGCAAGGTCTTCCTGGTTCC | GCTCCCTTACAGGCTGAGTTCT |
| CXCL1 | ATCGCACCCAAACCGAAGTC | TGGGGACACCTTTTAGCATCTT |
| CXCL2 | ATCCAGAGCTTGAGTGTGACG | GTTAGCCTTGCCTTTGTTCAG |
| CXCL5 | TTGATCGCTAATTTGGAGGTG | GCATTCCGCTTAGCTTTCTTT |
| CCL3 | ATGAAGGTCTCCACCACTGC | CTCAAGCCCCTGCTCTACAC |
| CCR2 | CCACACCCTGTTTCGCTGTA | TGCATGGCCTGGTCTAAGTG |
| CCR5 | ATTCTCCACACCCTGTTTCG | GTTCTCCTGTGGATCGGGTA |
| IFN-r | CGAAGCAGATGAATCCGCTGA | TGCGTGGAAATTGGGTGTCC |
| IL-15 | CATCCATCTCGTGCTACTTGTGTT | CATCTATCCAGTTGGCCTCTGTTT |
| IL-18 | ACAACTTTGGCCGACTTCAC | GGGTTCACTGGCACTTTGAT |
| CD69 | TGGTCCTCATCACGTCCTTAATAA | TCCAACTTCTCGTACAAGCCTG |
| Claudin | CGGGCAGATACAGTGCAAAG | ACTTCATGCCAATGGTGGAC |
| Occludin | ACCCGAAGAAAGATGGATCG | CATAGTCAGATGGGGGTGGA |
| Zo-1 | TGGGAACAGCACACAGTGAC | GCTGGCCCTCCTTTTAACAC |
| Eomes | GGCCCCTATGGCTCAAATTCC | GAACCACTTCCACGAAAACATTG |
| Gata3 | CCCCATTACCACCTATCCGC | CCTCGACTTACATCCGAACCC |
| Ets-1 | CCCTGGGTAAAGAATGCTTCC | GCTGATGAAGTAATCCGAGGTG |
| T-bet | AGCAAGGACGGCGAATGTT | GTGGACATATAAGCGGTTCCC |
| Perforin | TTGGTGGGACTTCAGCTTTCC | CCATACACCTGGCACGAACT |
| Granzyme B | ATGCTGCTAAAGCTGAAGAGT | TTCCCCAACCAGCCACATAG |
| IL-6 | TAGTCCTTCCTACCCCAATTTCC | TTGGTCCTTAGCCACTCCTTC |
| TLR4 | TGTTCTTCTCCTGCCTGACA | TGTCATCAGGGACTTTGCTG |
| TLR9 | GCTGTCAATGGCTCTCAGTTCC | CCTGCAACTGTGGTAGCTCACT |
| CXCL10 | GGATGGCTGTCCTAGCTCTG | TGAGCTAGGGAGGACAAGGA |
| CCL2 | TCCCAATGAGTAGGCTGGAC | TCTGGACCCATTCCTTCTTG |
| Acta2 | GTCCCAGACATCAGGGAGTAA | TCGGATACTTCAGCGTCAGGA |
| TGF-b | GTGGAAATCAACGGGATCAG | ACTTCCAACCCAGGTCCTTC |
| CDC20 | GCCCACCAAAAAGGAGCATC | ATTCTGAGGTTTGCCGCTGA |
| Ccnb2 | CAGAGAAAGCTTGGCAGAGG | TGAAACCAGTGCAGATGGAG |
| Ki-67 | GACAGCTTCCAAAGCTCACC | TGTGTCCTTAGCTGCCTCCT |

**Supplementary Table 2. Antibodies used in this study.**

|  | Antibody | Catalog no. | company |
| --- | --- | --- | --- |
| Human | APC-Cy™7 Mouse Anti-Human CD3 | 557832 | BD Pharmingen |
|  | PE/Cyanine7 anti-mouse/rat/human CD27 Antibody | 124216 | Biolegend |
|  | PE anti-human CD335 (NKp46) Antibody | 331908 | Biolegend |
|  | FITC anti-human CD16 Antibody | 302006 | Biolegend |
|  | Pacific Blue™ anti-human CD56 (NCAM) Antibody | 302520 | Biolegend |
|  | 7-AAD Viability Staining Solution | 420403 | Biolegend |
| Mouse | PE Mouse anti-mouse NK-1.1 | 561046 | BD Pharmingen |
|  | PE/Cyanine7 anti-mouse CD45 Antibody | 103113 | BioLegend |
|  | FITC anti-mouse CD3 Antibody | 100306 | BioLegend |
|  | APC/Cyanine7 anti-mouse/human CD11b Antibody | 101226 | Biolegend |
|  | Anti-mouse NKp46 (29A1.4) PerCP-Cy5.5 | 137610 | BioLegend |
|  | Anti-mouse CD27 (LG.3A10) APC | 124212 | BioLegend |
